# Kinetic Ultrasound-Assisted Extraction as a Sustainable Approach for the Recovery of Phenolics Accumulated through UVA Treatment in Strawberry By-Products

**DOI:** 10.3390/foods12162989

**Published:** 2023-08-08

**Authors:** Esteban Villamil-Galindo, Alejandro Gastélum-Estrada, Cristina Chuck-Hernandez, Marilena Antunes-Ricardo, Edwin E. Reza-Zaldivar, Andrea Piagentini, Daniel A. Jacobo-Velázquez

**Affiliations:** 1 Instituto de Tecnología de Alimentos, Facultad de Ingeniería Química, Universidad Nacional del Litoral, Santiago del Estero 2829, Santa Fe 3000, Argentinaampiagen@fiq.unl.edu.ar (A.P.); 2 Consejo Nacional de Investigaciones Científicas y Técnicas (CONICET), Santa Fe 3000, Argentina; 3 Tecnológico de Monterrey, Institute for Obesity Research, Av. Eugenio Garza Sada 2501, Monterrey 64849, Mexico; 4 Tecnológico de Monterrey, Escuela de Ingeniería y Ciencias, Campus Guadalajara, Av. General Ramón Corona 2514, Zapopan 45201, Mexico; 5 Tecnológico de Monterrey, Escuela de Ingeniería y Ciencias, Campus Monterrey, Av. Eugenio Garza Sada 2501, Monterrey 64849, Mexico

**Keywords:** bioactive compounds, green extraction, nutraceutical potential, Peleg’s model, ellagitannins

## Abstract

Ultrasound-assisted extraction (UAE) is an efficient and sustainable method for extracting bioactive compounds from agro-industrial by-products. Moreover, it has been reported that ultraviolet A (UVA) radiation can induce the biosynthesis and accumulation of bioactive phenolic compounds. This study optimized the efficiency of ultrasound-assisted extraction (UAE) for recovering ultraviolet A (UVA)-induced phenolic compounds in strawberry by-products (RF-N). The impact of three factors (solid-liquid ratio, ethanol concentration, and ultrasound power) on total phenolic compound (TPC) kinetics using Peleg’s model was investigated. The developed model showed a suitable fit for both RF-N and strawberry by-products treated with UVA (RF-E). The optimal UAE conditions obtained were of a 1:30 ratio, 46% ethanol, and 100% ultrasound power, resulting in an average yield of 13 g total phenolics kg^−1^. The bioaccessibility of phenolic compounds during in-vitro digestion was 36.5%, with agrimoniin being the predominant compound. UAE combined with UVA treatment increased the bioactivity of RF extracts, displaying significant anti-proliferative effects on HT29 and Caco-2 cancer cell lines, as well as anti-inflammatory potential and cellular antioxidant activity. The ultrasound proved to be a sustainable and effective technique for extracting phenolic compounds from RF, contributing to the valorization of strawberry agro-industrial by-products, and maximizing their nutraceutical potential.

## 1. Introduction

Within the Rosaceae family, the strawberry (*Fragaria x ananassa*, *Duch*.) is one of the main crops worldwide due to its vast consumption of fresh and derived products [1]. Due to their organoleptic, nutritional, and bioactive characteristics, strawberry consumption and production are growing, reaching around 9 million tons of strawberries cultivated worldwide annually [2]. Nonetheless, the high moisture content of strawberries makes them highly perishable, resulting in an expansive world market for their processing and industrial transformation. Postharvest processing of fruit and vegetables generates two types of by-products: avoidable and non-avoidable. Avoidable by-products are due to poor postharvest storage, processing, and transport handling. On the other hand, non-avoidable by-products are those agro-industrial by-products derived from the conditioning of fruits and vegetables where the non-usable or edible parts of the product are eliminated (peels, seeds, leaves, stalks, or fruit in non-adequate conditions) [3]. Both types of by-products (avoidable and non-avoidable) represent a major challenge in developed and developing countries regarding their management and disposal.

In the case of strawberry agro-industrial by-products (RF), this type of waste is considered non-avoidable and represents between 7–20% of production [4]. Various studies have been conducted to describe the composition of such by-products and their use in the food and nutraceutical industries, owing to their rich composition of bioactive compounds such as vitamins, amino acids, dietary fiber, and secondary metabolites, but mainly phenolic compounds [5,6]. In strawberries, the distribution of polyphenolic compounds depends on the tissue, variety, and crop-related factors. Nevertheless, ellagitannins, phenolic acids, and flavonoids such as anthocyanins and quercetin glycosides have been reported as the major phenolic compounds in the strawberry plant [7,8].

Obtaining health-promoting compounds from agro-industrial by-products is one strategy to revalue these discarded tissues [9,10,11]. These by-products can also serve as biofactories of phenolic compounds by applying different abiotic stresses (i.e., ultraviolet radiation, wounding, among others) that modulate their secondary metabolism, resulting in significant increases in the concentration of phenolic compounds [12]. In this context, we recently studied the effect of different ultraviolet A (UVA) conditions on the accumulation of phenolic compounds in strawberry agro-industrial by-products. The UVA-induced treatments resulted in a 240% increase in phenolic content. Specifically, the levels of ellagitannins, especially agrimoniin, were enhanced by approximately 300%. Moreover, the UVA treatment improved the bioaccessibility of all phenolic compounds present in the RF by 7%, thereby enhancing its potential as a source of nutraceutical compounds with anticancer, antioxidant, and anti-inflammatory properties [13]. Nonetheless, the efficient release and extraction of these phenolic compounds from the residues are necessary to propose their valorization as possible supplements or nutraceutical ingredients. It is worth noting that the solvation properties of RF phenolic compounds can vary depending on various factors that influence the solid–liquid extraction process, as discussed in previous reports [4,14].

Due to the structural diversity of phenolic compounds, no single system guarantees the total extraction of these compounds from different food matrices. Nevertheless, alternative technologies have been developed using environmentally friendly solvents with a lower toxicity. Process conditions have been optimized to match or even improve the yields of traditional technologies. For instance, Bustos-Hipólito et al. [15] reported that water at 100 °C extracted 25% more polyphenols than methanol at 20 °C. Moreover, binary hydroalcoholic mixtures have demonstrated good extraction yields in different plant matrices [16,17]. Solid–liquid extraction is a commonly employed method that utilizes traditional techniques such as maceration, percolation, and Soxhlet extraction. Nonetheless, these techniques often involve challenges such as high temperatures, extended extraction times, and the use of potentially toxic solvents. These limitations arise from the low effective mass transfer coefficient of these methods. To address these issues, alternative extraction technologies have been developed, including ultrasound-assisted extraction, microwave-assisted extraction, supercritical fluid extraction, pulsed electric fields, and pressurized liquid extraction. These technologies offer improved efficiency, shorter extraction times, and reduced environmental impact [18,19,20]. Shorstkii et al. [21] revealed that the application of pulsed electric fields resulted in an 8% improvement in the extraction yield of phenolic compounds from the kiwi peel. Nevertheless, Mohammad et al. [22] reported that pulsed electric fields’ technology had a negative impact on the phenolic compounds in apple juice, leading to their degradation. In the case of peach agro-industrial by-products, Plazzotta et al. [23] demonstrated that both microwave-assisted and ultrasound-assisted extraction methods yielded bioactive compounds with a low environmental impact. However, among the different techniques, ultrasound-assisted extraction stood out due to its low equipment complexity, versatility, and cost-effectiveness. As a result, ultrasound-assisted extraction emerges as a promising and viable technology for the extraction of bioactive compounds. Ultrasound-assisted extraction (UAE) employs sound waves with a frequency (~20 kHz) generated from transforming electrical energy into mechanical energy, which can propagate through gases or liquids [18]. This technique has been widely applied to extract phenolic compounds, and it has been demonstrated that using solvents such as ethanol and water can increase the extraction yield by up to 20% compared to traditional methodologies, depending on the plant matrix and its phenol profile [24]. Additionally, ultrasound-assisted extraction is a fast, sustainable, and easily scalable method at an industrial level [18].

The characteristics of the solvent, solute, and solid matrix, as well as factors such as extraction type and conditions, contact time, and temperature, among others, directly impact final yields and costs [25]. Therefore, it is essential to establish optimal process conditions through kinetic studies, applying mathematical models to optimize, simulate, and control solid-liquid extraction. Various mathematical models, including diffusion and empirical models, are employed in describing and predicting the mechanisms involved in the extraction kinetics of bioactive compounds. These models are widely documented in the literature [26,27,28].

Incorporating phenolic compounds from agro-industrial by-products in the human diet requires studying the behavior of these xenobiotics in human metabolism. The cytotoxicity of phenolic compounds has been studied in healthy cell lines with various metabolic functions [29,30] and in cell cultures of different types of cancer to determine their antioxidant and anti-inflammatory activity [31,32].

To the best of our knowledge, there have been no reports in the literature on the optimization of the extraction of UVA-induced phenolic compounds from strawberry agro-industrial by-products using (UAE) and on the in-vitro evaluation of the bioaccessibility and bioactivity of the obtained extracts. Therefore, the main objective of this study was to investigate the kinetic parameters of UAE of phenolic compounds from strawberry agro-industrial by-products and their stability during in-vitro digestion. Furthermore, the bioactive potential of the extracted compounds was evaluated in cellular models.

## 2. Materials and Methods

### 2.1. Reagents

Polyvinylpolypyrrolidone (PVPP), the Folin–Ciocalteu reagent, 2′,7′-dichlorodihydrofluorescein diacetate (DCFH-DA), pepsin from porcine gastric mucose (E.C.3.4.23.1 800–2500 units/mg protein), pancreatin from porcine pancreas (8XUSP), α-amylase, Insulin, dexamethasone (Dex), Oil Red-O (ORO), fluorescein, 2,2′azobis (2-amidinopropane) dihydrochloride (APPH), dichlorofluorescein diacetate (DCFH-DA), and lipopolysaccharides (LPS), acetonitrile and methanol (HPLC grade), ferulic acid, gallic acid, ellagic acid, procyanidin, quercetin, kaempferol, and albumin standards were obtained from Sigma Chemical Co. (St. Louis, MO, USA). DL-dithiothreitol (DTT) was purchased from Merck KGaA (Damnstadt, Germany). Sodium acetate trihydrate and potassium phosphate dibasic were acquired from Cicarelli Reagents S.A. (Santa Fe, Argentina). Fetal bovine serum and antibiotics for the cell culture were from GIBCO (Grand Island, NY, USA). The cell titer 96 aqueous one solution cell proliferation assay and Griess reagent were obtained from Promega (Madison, WI, USA). The 3-isobutyl-l-methylxanhine (IBMX) was obtained from Galbiochem (San Diego, CA, USA).

### 2.2. Plant Material

The strawberry agro-industrial by-products (RF-N) (*Fragaria x ananassa Duch*) consist of sepals, peduncles, and fruit remnants. The RF-N were obtained during postharvest industrial processing of strawberries from a field in Coronda, Argentina (31°58′00″ S 60°55′00″ W). To enhance the content of phenolic compounds, RF-N was subjected to a UVA radiation dose of 86.4 KJ m^−2^. The UVA treatment was conducted in a climate chamber (Memmert, Germany) equipped with two 8 W UVA lamps emitting light within the spectrum range of 320–400 nm. The intensity of UVA radiation was determined using a UVA dosimeter (model 501, Solar Light Co., Glenside, PA, USA) and the compounds were stored at 15 °C for 46 h to induce the biosynthesis of phenolic compounds as previously described by Villamil-Galindo et al. [13]. The strawberry agro-industrial by-products non-treated with UVA will be referred to as RF-N, while those treated with UVA will be referred to as RF-E. The RF-N and RF-E samples were packed in 40 µm polypropylene bags and stored at −80 °C. Before each extraction assay, the frozen strawberry by-product was ground in a mortar using CO_2_ (particle size ≤ 1 mm).

### 2.3. Experimental Design, Modelling, and Optimization of Phenolic Compound Extraction

The statistical tool used to evaluate the interaction between different variables and identify the optimum ultrasound extraction process conditions was the response surface methodology (RSM), specifically the Box–Benkhen (BB) design. The optimization design was evaluated on RF-N only and then validated for RF-E. A total of three parameters with three levels were used: ethanol concentration (0, 40, 80%), solid-liquid ratio (1:20, 1:30, 1:40 g mL^−1^), and percentage of ultrasound (US) power (20, 60, 100%). A total of 15 experiments, including two replicates at the center point, were performed, and the corresponding coded levels were used (X1, X2, X3) in the model equations: low (−1), middle (0), and high (+1). For each BB design point, the total phenolic content (TPC) was determined during the 24 min of extraction from the RF (according to previous assays).

The TPC obtained during UAE from the RF, for each BB design point, were fit to an empirical Peleg’s model [Equation (5)] due to the similar shape of the sorption and extraction curves [20], and the kinetics parameters (K1 and K2; Equation (5)) were determined through non-linear regression.

(1) $$Y\left(t\right)=\frac{t}{\left(\frac{1}{K1}\right)+\left(\frac{1}{K2}\right)*t}$$

where *t* is the extraction time (min), *Y(t*) is the extraction yield at time *t* (g GAE Kg^−1^ RF), K1 is the Peleg’s rate constant (min^−1^), and K2 is the Peleg’s capacity constant (g GAE Kg^−1^). Through the experimental design, the constants of the Peleg’s model were optimized.

Classical second-order polynomial equations were used to describe the effect of extraction parameters on each kinetic constant, Ki; Equation (2).

(2) $$Ki=\beta_{0}+\sum_{i=1}^{3}\beta_{i}X_{i}+\sum_{i=1}^{2}*\sum_{j=2.j>i}^{3}\beta_{ij}X_{i}X_{J}+\sum_{i=1}^{3}\beta_{ii}X_{i}^{2}$$

where Ki is the Peleg’s model constants K1 or K2, $\beta_{0}$ is the model constant, $\beta_{i}$ is the linear coefficient, $\beta_{ii}$ is the quadratic coefficient, $\beta_{ij}$ is the coefficient for the interaction effect, and X is a dimensionless coded value of the independent variables.

The models developed for K1 and K2 were used to obtain the extraction parameters’ values that maximize Peleg’s model constants. To determine the concordance between calculated and experimental data, the adjusted coefficient of determination (adj-R^2^), and the root mean square error (RSME; Equation (3)), were used.

(3) $$RSME=\frac{\sqrt{\left(\frac{1}{n}\right)\sum_{p=1}^{n}*({exp}_{i}-{mod}_{i}{)}^{2}}}{{Exp}_{max}}$$

To validate the models, two extractions were performed at the optimal parameters obtained with RF-N and RF-E tissues. Furthermore, the changes in TPC over time were modelled using Equation (1) (obtaining K1 and K2), and comparing the kinetic parameters obtained from Equation (2) at the optimal Xi values. In addition, individual phenolic compound profiles were determined for strawberry by-products with and without UVA treatment under the previously determined optimal conditions. These results were also compared with a control extraction carried out under optimal Ratio (X1) and ethanol concentration (X2) values but without ultrasound assistance (MCE) in RF-N and RF-E. The MCE extraction process involved contacting the ground frozen RF tissue with the extraction solvent at the optimum extraction ratio determined. This contact was maintained for 24 min at room temperature without any shaking. During this period, different samples were collected at specific time intervals to determine the kinetic constants of Equation (1).

### 2.4. Ultrasound-Assisted Extraction (UAE) Procedure

The UAE technique used an ultrasonic probe system (UP400S Hielscher Inc., Teltow, Germany) with a 22 mm diameter cylindrical sonotrode (Horn 22, Hielscher Inc., Teltow, Germany). The equipment’s power was controlled between 20–100% of total power (400 W), with a frequency of 24 kHz. Table 1 shows the calculation of the ultrasonic intensity applied for each type of solvent and the percentage of power used, according to Equations (4) and (5), determined by the calorimetric method by monitoring the temperature increase due to the conversion of acoustic energy into heat energy [19].

(4) $$P=mC_{p}\frac{dT}{dt}$$

where P is the ultrasound (US) Power, dT/dt represents the temperature rise per second, Cp is the heat capacity of different solvents employed at constant pressure (J Kg^−1^ °C^−1^), and m is the mass of the solvent (Kg).

Equation (5) was used to calculate the applied ultrasonic intensity.

(5) $$UI=\frac{4P}{\pi D^{2}}$$

where the ultrasonic intensity is UI (W m^−2^), and the US power is P in W according to Equation (4). The internal diameter (m) at the tip of the probe is D.

For each extraction, the ground strawberry by-products were mixed with the selected solvent in the appropriate ratio. The mixture was then sonicated continuously for 24 min using an ultrasonic sonicator. After sonication, the mixture was centrifuged at 12,000× *g* for 20 min at 4 °C using a Neofuge 18 R Heal Force centrifuge (Shanghai, China). The resulting supernatant was carefully separated and reserved for further analysis of the extracted phenolic compounds. The temperature was monitored using a thermocouple coupled to the ultrasonic probe system, and no temperature control or agitation was used during extraction. Samples were taken at 3, 6, 9, 12, 15, 18, 21, and 24 min to study the extraction kinetics from RF. The extracts were then centrifuged for 10 min at 10,000× *g* min^−1^ (Centrifuge 5804R, Eppendorf, Hamburg, Germany), and the supernatant was stored at −20 °C until analysis.

### 2.5. Total Phenolic Content (TPC)

The TPC of the RF extracts was determined using the Folin–Ciocalteu method [33]. Briefly, 20 µL of the RF extracts were mixed with 100 µL of Folin–Ciocalteu reagent, 100 µL of sodium carbonate solution (10%), and 780 µL of distilled water. The mixture was incubated at 37 °C for 30 min in triplicate, then transferred to a 96-well plate, and the absorbance was measured at 765 nm using a microplate reader (Synergy HT, Bio-Tek, Winooski, VT, USA). The TPC results were expressed as g of gallic acid equivalents (GAE) per Kg of strawberry by-product (g GAE Kg^−1^).

### 2.6. Phenolic Compound Determination by High-Performance Liquid Chromatography (HPLC-DAD)

For the high-performance liquid chromatograph (HPLC) coupled to a Diode-Array Detector (DAD) (HPLC-DAD) analyses, the extracts obtained as described in the previous sections were dried using a rotatory vacuum evaporator (EZ-2.3, Genevac Ltd. Ipswich, UK) and stored at −80 °C until analyzed. A high-performance liquid chromatograph coupled to a Diode-Array Detector (1200 Series, Agilent Technologies, Santa Clara, CA, USA) was employed to perform the phenolic compound profile study. Separation was carried out using a 250 × 4.6 mm Supelco 5 µm LC-18 reversed-phase column. Before analysis, samples were resuspended in 500 µL of 50% methanol. The analysis of phenolic compounds was conducted following the procedure described by Villamil-Galindo et al. [14]. Concisely, the linear gradient consisted of 1% formic acid (A) and acetonitrile (B) in the following proportions: 90–75% of (A) for 30 min, followed by 75–40% of (A) for 30–45 min, at a flow rate of 1 mL min^−1^ at 25 °C. Before analysis, the resuspended samples were filtered through a 0.45 μm filter (Gamafil, Buenos Aires, Argentina), stored at 4 °C, and protected from light. The identification of phenolic compounds was based on retention times and UV-Vis absorption spectra in relation to common standard phenolic compounds in strawberries. The calibration curves of gallic acid (GA) (0.002–2 mg mL^−1^, R^2^ 0.974), coumaric acid (CUA) (0.002–2 mg mL^−1^, R^2^ 0.985), ferulic acid (FRA) (0.002–2 mg mL^−1^, R^2^ 0.984), quercetin (QCN) (0.002–2 mg mL^−1^, R^2^ 0.983), and ellagic acid (EA) (0.002–2 mg mL^−1^, R^2^ 0.989) were used for quantification purposes. The concentrations of phenolic compounds in the extracts were calculated and expressed as g of phenolic compound per Kg of RF (g Kg^−1^), and the arithmetic sum of the individual phenolic compounds was reported as the total phenolic compounds determined by HPLC (TPC_HPLC_).

### 2.7. In-Vitro Gastrointestinal Digestion Assay

In-vitro digestion tests aim to simulate the conditions of in vivo digestion to study the interaction of food matrices, drugs, or bioactive compounds under simulated physiological conditions and determine their bioaccessibility. The assay followed the methodology proposed by Flores et al. [34] consisting of three stages: the oral phase using Simulated Saliva Fluid (SF), the gastric phase using Simulated Gastric Fluid (GF), and the intestinal phase (IF) using Simulated Duodenal Fluid (DF) and bile solution (BF). The composition of each phase was prepared according to Table S1 in the Supplementary Material.

Optimal extracts (USRF-N and USRF-E) were freeze-dried (Labconco, Kansas City, MO, USA) to 5.2% moisture content and ground to a particle size of <1 mm. After each phase, aliquots of 0.5 mL were collected, immediately heated in a water bath at 90 °C for 3 min to inactivate the enzymes, and centrifuged at 4 °C for 15 min at 9000× *g* (Centrifuge 5804R, Eppendorf, Hamburg, Germany). The supernatant was recovered, and each digestion was performed in triplicate. HPLC-DAD was used to analyze each phase, and the final phase (intestinal phase) was used for cell culture assays. The results were expressed as a percentage (%) of bioaccessibility according to the ratio between the phenolic compound concentration in the intestinal fraction and their initial concentration in the raw material or dried extract, as described in Equation (6).

(6) $$Bioaccessibility\;\left(\%\right)=\frac{Phenolic\;compounds\;in\;GF\;or\;IF}{Phenolic\;compound\;in\;raw\;material\;\left(Dried\;extract\right)}\times 100$$

### 2.8. Cell Culture

Cell lines, Human colorectal adenocarcinoma cells (Caco-2 and HT29), mouse macrophage (RAW 264.7), 3T3-L1 mouse adipocytes, and primary dermal fibroblasts: normal, human, adult cells (HDFa), were purchased from the American Type Culture Collection (ATCC) (Manassas, VA, USA). The cells were maintained in Dubelcco’s Modified Eagle Medium (DMEM) supplemented with 5% fetal bovine serum (FBS) and 1% antibiotic, streptomycin (10,000 µg mL^−1^), at 37 °C and 5% CO_2_ in a humidified incubator.

#### 2.8.1. Anti-Inflammatory Potential

Nitric oxide (NO) is a free radical that plays various biological roles, particularly when released by macrophages, astrocytes, and microglia in response to proinflammatory signals. Due to their low concentrations and short half-life, a direct NO measurement is challenging in biological systems. Therefore, stable metabolites such as nitrites and nitrates are commonly quantified as indicators of NO production [35]

The resuspended digestion of freeze-dried ultrasound-assisted extraction (UAE) extracts was used to assess their anti-inflammatory and cytotoxicity potential (cell viability higher than 80%) at different concentrations (0.11–1.75 mg mL^−1^). The RAW 264.7 cell line measured NO production induced by LPS from *Salmonella enterica* serotype typhimurium. The assay followed the procedure outlined by López-Barrios et al. [35] with some modifications. Initially, 100 µL containing 5 × 10^5^ cells mL^−1^ were seeded in each well of a 96-well cell culture plate. After 24 h, triplicates of 50 µL samples were added in a previously determined safe concentration of 1.75 µg/mL of digested fraction, and then, incubated for 4 h at 37 °C. Subsequently, 50 µL of LPS (10 µg mL^−1^) was added to half of the wells to induce the inflammatory process, while the other half served as the control with 50 µL of the DMEM medium. The plate was then incubated for 18 h at 37 °C. NO production was determined using the Griess Reagent System (Promega, Madison, WI, USA) following the manufacturer’s instructions. The results were expressed as the % of inhibition relative to the positive control plate and cell viability using the Cell Titer 96 Aqueous One Solution Cell Proliferation Assay, according to the manufacturer’s instructions.

#### 2.8.2. Cellular Antioxidant Activity

The level of reactive oxygen species (ROS) produced by cells in the presence of digested extracts of USRF-N and USRF-E (0.23 mg mL^−1^) was measured following the methodology proposed by Wan et al. [36] with certain modifications as described by [13]. Fluorescence emission at 538 nm (excitation at 485 nm) was measured every 2 min for 120 min at 37 °C. The results were expressed following Equation (9).

(7) $$\%\left(CAA\right)=1-(\int SA/\int CA)$$

where ∫SA is the integrated area under sample fluorescence versus the time curve and ∫CA is the integrated area from the control curve.

#### 2.8.3. Anti-Proliferative Activity

The effect of digested optimal UAE from UVA-treated RF (USRF-E) and its control (USRF-N) on the viability of Caco-2, HT29, and HDFa cells was tested using the Cell Titer 96 Aqueous One Solution Cell Proliferation Assay. The cell viability was determined following that reported by Pacheco-Ordaz et al. [37]. Different digested UAE extracts’ concentrations were tested (0.0094–122 mg mL^−1^). The IC_50_ values (half maximal inhibitory concentration) for each sample were determined from the percentage viability versus concentration data; these were fitted to an asymmetric sigmoidal equation, and the appropriate concentration to inhibit 50% viability in the cells studied was calculated.

#### 2.8.4. Anti-Obesogenic Potential

Murine cell line 3T3-L1 (ATCC) was cultured under standard conditions to evaluate the anti-obesogenic potential of RF extracts. Cells were maintained in DMEM with 10% NBCS until confluence, changing the medium every 48 h. The Cell Titer 96 Aqueous One Solution Cell Proliferation Assay confirmed non-toxic dilutions for the 3T3-L1 differentiation process. Cells (10 × 10^3^ cells/well) were subcultured in a 96-well microplate for 24 h, followed by 24 h treatment. Triplicate treatments were performed. Cells (8000 cells per well) were then seeded in 24-well plates and differentiated using DMEM with 10% FBS, 0.5 mM IBMX, 1 µM Dex, and 10 µg mL^−1^ insulin. After four days, medium changes were made using DMEM with 10% FBS and 10 µg mL^−1^ insulin. Digested RF (3.05 mg mL^−1^) fractions were suspended in 0.01 M PBS pH 7.4. Samples were added to the cell plate, along with the induced differentiation solution in triplicate. After eight days, Oil Red O (ORO) staining was performed to determine adipocyte lipid accumulation. Cells were fixed with 4% paraformaldehyde during 15 min, permeated with 60% isopropanol for 15 s, stained with ORO reagent (5 mg mL^−1^) for 20 min, and then, observed under a microscope. After removing the ORO solution and washing cells with PBS, the ORO stain was solubilized in 60% isopropanol and transferred to a 96-wells plate; total lipid content was measured at a 490 nm wavelength, calculating the percentage relative to the control.

### 2.9. Statistical Analysis

The kinetic constants of Peleg’s model (K1 and K2) were determined through non-linear regression using the STATGRAPHICS Centurion XV software (StatPoint Technologies Inc., Warrenton, VA, USA). The BB design was conducted and analyzed using Design Expert software version DX 7.1.3 (Stat Ease Inc., Minneapolis, MN, USA). It was also used to obtain the response surface and linear contour figures. The predicted coefficients were examined based on F-values and *p*-values. The Derringer’s desirability function was employed to perform the optimization of multiple responses analyses performed to optimize the kinetic extraction parameters of total phenolic compounds. Moreover, it included an analysis of variance (ANOVA) and regression analysis. Triplicate treatments were conducted, and the Tukey’s test and t-tests (*p* < 0.05) were performed to determine significant differences among the means of treatments, using STATGRAPHICS Centurion XV software (StatPoint Technologies Inc., Warrenton, VA, USA).

## 3. Results and Discussion

### 3.1. Influence of Extraction Variables on the Total Phenolic Content (TPC) and Its Peleg’s Model Kinetic Parameters

Based on previous analyses, the key factors that significantly influence the extraction of phenolic compounds from strawberry agro-industrial by-products (RF) were identified in the current study. As a result, the impact of three variables was investigated: the extraction ratio (X1), the percentage of ethanol in the extraction solution (X2), and the percentage of ultrasonic probe power (X3). These factors were explicitly selected for examination due to their potential influence on the extraction process and the recovery of phenolic compounds from RF. Table 2 presents the values of the independent variables of the experimental design conducted and the constants of Peleg’s model. Each run of the experiment involved the determination of the variation of TPC during the extraction time up to 24 min. The TPC obtained from each run were then fitted to the Peleg’s model (Equation (1), adj-R^2^ between 0.96–0.99), allowing for calculating the kinetic parameters (K1 and K2) associated with each run of the Box–Behnken (BB) design.

**Figure 1:**
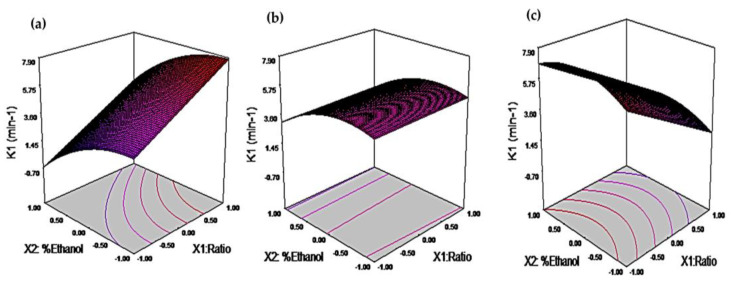
Response surface plots of the rate constant of the kinetic Peleg’s model (K1). (**a**): 20%US. (**b**): 60%US. (**c**): 100%US.

The TPC extraction rate constant (K1) from the Peleg model was found to be significantly affected (*p* < 0.05) by the variation of the ratio, ethanol concentration, and percentage of ultrasound power. Notably, the interaction between ratio ∗ percentage of ultrasound power, and ethanol concentration ∗ percentage of ultrasound power had a significant (*p* < 0.05) impact on K1 (Supplementary Material Table S2). The extraction ratio (X1) plays a crucial role in controlling the driving force in the mass transfer phenomenon by creating a concentration gradient between the strawberry agro-industrial residues (RF) and the extraction solvent. As observed in Table 2, increasing X1 from a ratio of 1:20 to 1:40 (*w/v*) while keeping ethanol concentration (X2) constant at 40% ethanol and X3 at 20% ultrasonic power resulted in a 127% increase in the extraction rate constant (K1). Nevertheless, when X1 was increased along with X2 (40–80% ethanol) while maintaining X3 at 20% ultrasonic power, the increase in K1 was only 21% (Figure 1).

The solubility of phenolic compounds can vary depending on their chemical nature and the extraction conditions employed. In the context of solid–liquid extraction, the interactions between the solvent and solute and solvent–solvent interactions play a crucial role in determining extraction efficiency [4]. Ethanol, a polar protic solvent, can donate hydrogen bonds through its hydroxyl group. Nevertheless, it has a moderate polarity, as indicated by its solvatochromic index (E^nT^) of 0.65. This moderate polarity limits its capacity to extract highly polar phenolic compounds efficiently. Mixtures incorporating a more polar solvent, such as water (E^nT^ = 1), can enhance the extraction of a broader range of phenolic compounds. By using mixtures such as 80% ethanol (E^nT^ = 0.69) and 40% ethanol (E^nT^ = 0.73), a broader spectrum of phenolic compounds can be effectively extracted [38]. The power of the ultrasonic probe played a significant role in the extraction rate of TPC. This effect is evident in the extraction experiments conducted with a fixed extraction ratio of 1:20 (X1), 40% ethanol (X2), and varying levels of ultrasonic probe power (X3) ranging from 20% to 100% US, leading to a 135% increase in the K1 parameter of the extraction system (Figure 1).

This finding highlights acoustic energy’s impact on TPC extraction efficiency. Different combinations of extraction variables can influence the extraction rate of TPC. Thus, finding the optimal point for efficient extraction of phenolic compounds from RF is crucial. The maximum TPC extraction capacity of the extraction system, as determined by the K2 parameter in Peleg’s model, was significantly influenced by the interactions between ratio∗ethanol concentration, ratio∗percentage of ultrasound power, and ethanol concentration∗percentage of ultrasound power (Supplementary Material, Table S2). This was confirmed by the analysis of variance, which revealed the highest F-values ranging from 16.6 to 46 and the lowest *p*-values ranging from 0.007 to 0.027.

**Figure 2:**
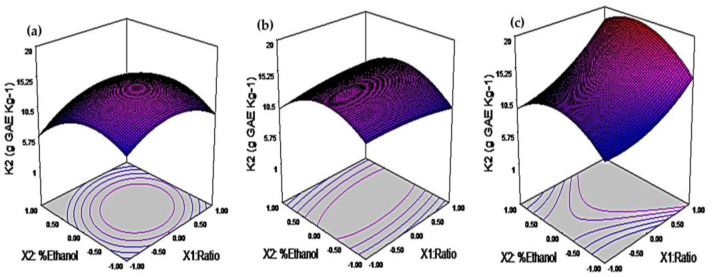
Response surface plots of the capacity constant of the kinetic Peleg’s model (K2). (**a**): 20%US. (**b**): 60%US. (**c**): 100%US.

In the experimental runs of the BB design, the TPC obtained from the RF ranged from 7.1 to 13.5 g GAE kg^−1^ RF. The K2 parameter, adjusted to the experimental data, ranged from 9.6 to 19.3 g GAE kg^−1^ RF. When the ratio increased from 1:20 to 1:40 (*w/v*), the percentage of ultrasound power increased from 20% to 100%, and the ethanol concentration was kept constant at 40%; the K2 value increased about 53% (Figure 2, Table 2). This can be attributed to the higher extraction ratio, which creates a greater concentration gradient and a stronger driving force. The increase in ultrasonic power leads to the generation of acoustic energy, causing the cavitation phenomenon, which enhances the disruption of the plant matrix, facilitating the interaction with the solvent and improving the solubilization of the TPC [19]. On the other hand, when ethanol concentration increased from 0% to 80% and X3 from 20% to 100% US, while X1 remained constant at 1:30, there was no significant effect on K2, which changed from 12.5 to 12.1 g GAE Kg^−1^. This result suggests that high ethanol concentrations in the extraction solution did not favor the recovery of phenolic compounds present in RF.

#### 3.1.1. Modelling of Ultrasound-Assisted Extraction (UAE) Kinetic Parameters

The kinetic parameters of Peleg’s model, K1 and K2, were determined for each experimental extraction of TPC from RF. These parameters were then adjusted to a quadratic model (Equation (2)), further reduced by considering only the significant coefficients. The adjusted models are represented by Equations (8) and (9). The high adj-R^2^ values determined (0.86–0.97) indicate that a substantial proportion of the total variation in K1 (86%) and K2 (97%) can be attributed to the factors’ ratio, ethanol concentration, and percentage of ultrasound power and their interactions.

(8) $$K1=5.06+0.055X1-0.86X2+0.43X3-2.19X1X3+1.09X2X3-1.03{X2}^{2}$$

(9) $$K2=14.35+0.14X1+0.051X2-0.30X3+0.83X1X2+1.39X1X3-1.12X2X3-0.53{X1}^{2}-3.27{X2}^{2}\;+0.093{X3}^{2}+2.24{X1}^{2}X3+1.91X1{X3}^{2}$$

The quadratic model allows for a comprehensive understanding of the relationship between these factors and the kinetic parameters of TPC extraction. By analyzing the coefficients of the reduced model, it is possible to identify the most influential factors and their interactions, providing valuable insights for optimizing the extraction process and maximizing TPC yields. The models show that the rate (K1) of TPC extraction from RF was higher when using a higher extraction ratio (1:40 *w/v*) and lower power of the ultrasonic probe combined with 100% water as the extraction solvent (Figure 1). Interestingly, adding 40% ethanol to the extraction solution did not result in a higher extraction rate, even with higher ultrasonic probe power. Water, a good swelling agent, can efficiently diffuse through the plant matrix, aiding extraction. Nonetheless, its high surface tension (105 cal mol^−1^ Å^−2^ at 25 °C) and its viscosity of 0.89 cP allow for a higher ultrasonic intensity according to Table 2 of 316,666.7 W m^−2^. Nevertheless, these conditions did not favor the extraction rate [39,40].

Additionally, the high polarity of water makes it less effective in interacting with low to medium-polarity metabolites present in RF [4]. On the other hand, ethanol, when added to the extraction solution, reduces the surface tension due to its moderate surface tension (31.62 cal mol^−1^ Å^−2^ at 25 °C), and the increase in viscosity when mixed with water (1.9 Cp) lowers the UI to a range between 129,973.05–208,713.49 W m^−2^, which favor the rapid solvation of TPC, especially in the case of 40% ethanol [39,41]. Furthermore, in solutions with similar concentrations of water and ethanol, water acts as a swelling agent that facilitates the penetration of the plant matrix, while ethanol aids in solvating a wider range of phenolic compounds. Interestingly, it was observed that the extraction rate was slower when the ethanol concentration in the extraction solution was increased to 80% compared to 40% ethanol. This result suggests that higher ethanol concentrations do not improve phenolic compounds’ extraction from RF (Figure 1).

According to Equation (9), the extraction capacity of the system (K2) follows a similar trend as K1, increasing with an increase in the extraction ratio and the percentage of ultrasonic (US) power. The maximum extraction capacity is achieved when the ethanol concentration in the extraction solution is 40%. It is worth noting that the physicochemical properties of the water:ethanol 60:40 (*v/v*) mixture contribute to the higher extraction capacity observed, reaching up to 19.3 g GAE kg^−1^. This significant extraction capacity is attributed to the interaction among the extraction ratio, percentage of US power, and ethanol concentration. These findings are further supported by the response surfaces and contour lines provided in Figure 2. The figures demonstrate that an increase in the percentage of US power and extraction ratio leads to an enhancement in the extraction capacity of the system, validating the relationship described by Equation (1).

#### 3.1.2. Optimization of RF Phenolic Compounds Ultrasound-Assisted Extraction (UASE)

Equations (8) and (9) were used for the simultaneous optimization of the kinetic parameters in the Peleg model. This approach aimed to maximize the extraction rate (K1) and the extraction capacity (K2) of TPC from RF. By obtaining these optimal kinetic constants, a unified model was developed to predict TPC extraction yield over time, considering the three process variables studied.

The optimized model obtained through this approach represents a valuable tool for a more efficient extraction process for recovering high-value secondary metabolites from strawberry agro-industrial by-products. It provides the ability to predict how various process factors influence extraction yields, allowing for the determination of the minimum extraction time required to achieve maximum ultrasound-assisted extraction. The Derringer desirability function was employed to perform the optimization of multiple responses. The optimized conditions include an extraction ratio of 1:30 g mL^−1^, an extraction solvent composed of 46.4% ethanol, and the application of 100% ultrasound power intensity (400 W). Under these process conditions, maximum values for the kinetic parameters K1 (5.83 min^−1^) and K2 (14.35 g GAE Kg^−1^) were obtained and had a desirability coefficient of 0.73.

**Figure 3:**
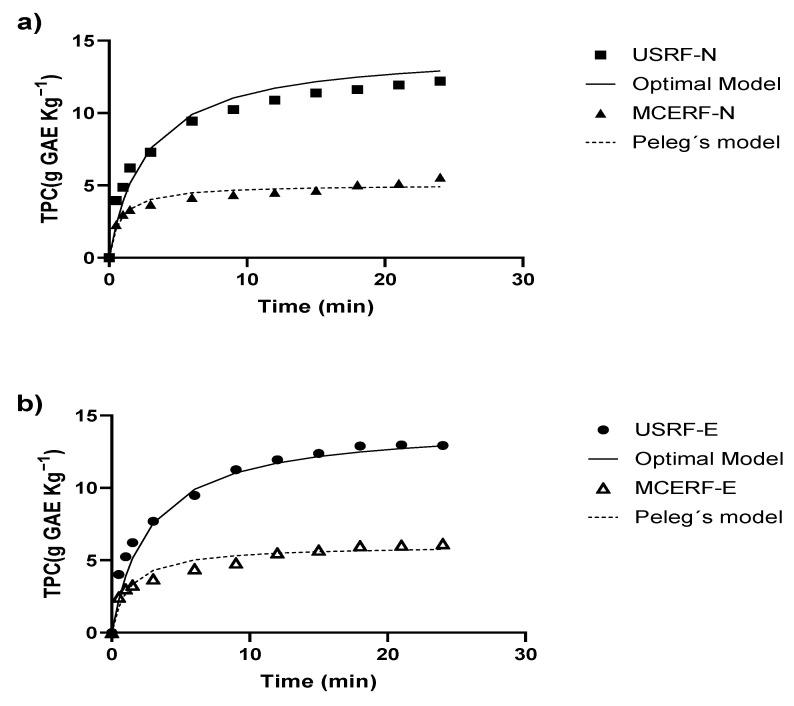
Experimental and predicted total phenolic content during extraction at optimal process conditions with ultrasound-assisted (US) and control maceration extraction (MCE) from (**a**) Strawberry by-products (RF-N); (**b**) UVA-induced biosynthesis of phenolic compound (RF-E).

To validate the models obtained, the extraction of phenolic compounds was performed at optimal conditions from two tissues: RF treated with UVA (USRF-E) and RF without the UVA process (USRF-N). The aim was to investigate whether the predicted models could be applied to both tissues. To evaluate the efficiency of UAE, an extraction process was also conducted using the optimal process parameters but without the use of acoustic energy from the ultrasonic probe. This extraction method, known as maceration extraction (MCE), was applied to both the UVA-treated RF tissue (MCERF-E) and the control RF tissue (MCERF-N). Figure 3 illustrates the extraction of TPC over 24 min at the optimal process conditions. In Figure 3a, the extraction results for USRF-N and MCERF-N are shown.

Figure 3 reveals a significant difference in extraction yields between ultrasound-assisted extraction and maceration. From the third minute of extraction onwards, the ultrasound-assisted extraction yields were consistently higher than maceration. Specifically, the extraction yields for USRF-N were 123% higher than MCERF-N, while the extraction yields for USRF-E were 111% higher than MCERF-N. These differences in yields can be attributed to the extraction efficiency of the respective methods.

Table 3 provides additional information on the maximum concentration of TPC reached during the extraction process. For USRF-N, the maximum concentration obtained was 11.4 g GAE Kg^−1^, while for MCERF-N, it was only 5.1 g GAE Kg^−1^. Similarly, for USRF-E, the maximum concentration reached was 12.9 g GAE Kg^−1^, whereas, for MCERF-E, it was 6.1 g GAE Kg^−1^. These values further demonstrate the higher efficiency of ultrasound-assisted extraction in achieving higher TPC concentrations than the maceration extraction. The results obtained from the optimal model (Equations (1), (8) and (9)) were consistent with the experimental data, confirming its predictive capability. The predicted TPC extraction yield of 12.2 g GAE Kg^−1^ closely matched the experimental values obtained for USRF-N and USRF-E. As expected, the TPC extraction yields were higher for RF treated with UVA. Regarding the kinetic parameters, the predicted (Equations (1) and (8)) velocity parameter K1 was lower (5.83 min^−1^) compared to the determined (Equation (1)) from experimental TPC values (7.7 min^−1^ for USRF-N and 7.4 min^−1^ for USRF-E). This result indicates that the actual rate of TPC extraction under these extraction conditions was higher than those predicted by the model, even surpassing the extraction efficiency of MCE.

These results highlight the effectiveness of UAE extraction in achieving a higher extraction rate of TPC. The values obtained from the optimal model were obtained using the Equations (1), (8) and (9), consistent with the experimental data fitted to Equation (1), confirming its predictive capability. For the extraction capacity parameter K2, the predicted value was 14.4 g GAE Kg^−1^, similar (*p* > 0.05) to the experimental value (13.8 g GAE Kg^−1^) determined for USRF-E. In all cases, the values obtained using UAE were higher than those achieved with MCE. This result further emphasizes the higher capacity of UAE for extracting a higher concentration of phenolic compounds from RF. According to Figure 3, after 15 min of extraction, the variation in TPC was not significant. Thus, it can be estimated that the maximum yield can be obtained under optimal process conditions after 15 min of extraction.

The temperature is an essential factor influencing the extraction efficiency in UAE processes. In the present study, the temperature during the extraction process was not controlled, ranging from 64 to 82 °C with a mean temperature of 68 °C for 40% ethanol and 100% US power. Working at higher temperatures can have several effects on the extraction process. It promotes the diffusion of phenolic compounds through the solvent, allowing for faster extraction kinetics, which could be detrimental for thermosensitive compounds that may degrade or undergo structural changes at higher temperatures. Furthermore, the increased temperature enhances the solvation of phenolic compounds, leading to a higher extraction yield [20].

Studies conducted by Galván D’Alessandro et al. [25] on *Aronia melanocarpa* residues reported a maximum recovery of total phenolic compounds (TPC) and anthocyanins at 70 °C using 100 W ultrasonic power and 50% ethanol as the extraction solvent. The reported values were 60 g Kg^−1^ for TPC and 12 g Kg^−1^ for anthocyanins. According to Urango et al. [20], thermosonication was reported to be up to 50 times cheaper than conventional technologies. This cost advantage can be attributed to several factors. The thermosonication generally requires shorter extraction times than traditional methods, reducing energy consumption and operational costs. Additionally, an ultrasound can enhance extraction efficiency, allowing for higher yields and reducing the solvent required, which can result in cost savings related to the purchase and disposal of solvents.

The composition of agro-industrial by-products from strawberries has demonstrated excellent potential as a source of phenolic compounds obtained through UAE. The yields obtained from RF through UAE were higher than the TPC content reported for wild strawberry plant leaves extracted by maceration with 100% water at 25 °C (9.2 g GAE Kg^−1^) [42]. Compared to other agro-industrial residues, such as pomegranate peel and grape seeds, the TPC content obtained from strawberry agricultural by-products through UAE was also higher. Pomegranate peels that were extracted with the ultrasound at 25 °C for 60 min yielded a TPC content of 1.4 g GAE Kg^−1^, while the yield of TPC extracted from grape seeds using UAE at 56 °C with 50% ethanol for 29 min was 5.4 g GAE Kg^−1^ [43,44].

### 3.2. Phenolic Compounds Profile

**Figure 4:**
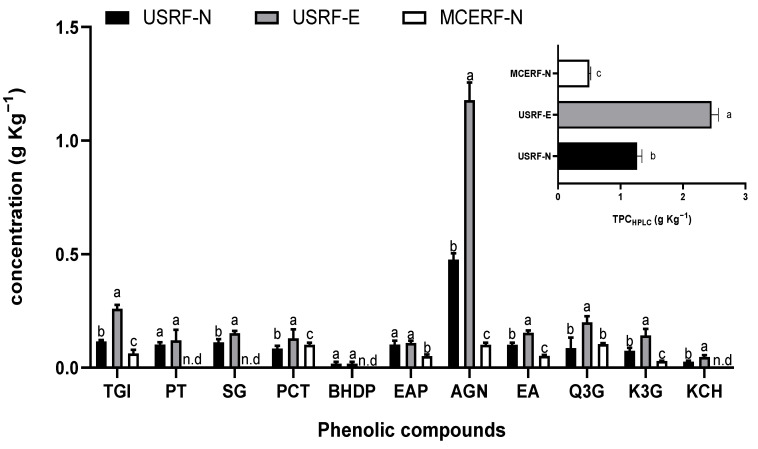
Individual and total phenolic compound profile for the different extraction processes of RF. TGI, tetragalloyl glucose isomer; PT, procyanidin trimer; SG, saguinn; PCT, procyanidin tetramer; BHDP, bis-HHDP-glucose isomer; EAP, ellagic acid pentoxide; AGN, agrimoniin; EA, free ellagic acid. procyanidin tetramer; Q3, quercetin 3-o-glucuronide; K3G, Kaempferol 3-o-glucuronide; KCH, kaempferol-coumaroyl hexoxide; P3G, pelargonidin-3-o-glucuronide; TPC_HPLC_, Total phenolic content by HPLC. n.d., Not detected. Different lowercase letters indicate significant differences (*p* < 0.05) among the different extraction methods and RF conditions, as determined by Tukey’s test.

The Ultrasound assisted extraction (UAE) allowed the extraction, identification, and quantification of eleven main phenolic compounds, headed by ellagitannins such as tetra galloyl glucose isomer (TGI), sanguiin-H6 (SG), Bis-HHDP-glucose isomer (BHDP), and agrimoniin (AGN, the main compound in RF); Procyanidin derivatives such as procyanidin trimer (PT) and procyanidin tetramer (PCT); Ellagic acid derivatives including ellagic acid pentoxide (EAP) and free ellagic acid (EA); and the Flavonols like quercetin-3-o-glucuronide (Q3G), kaempferol-3-o-glucuronide (K3G), and kaempferol coumaroyl hexoxide (KCH). The different parts of the strawberry plant present in the RF tissue allow for various phenolic compounds with different bioactive properties. As shown in Figure 4, USRF-E had the highest concentration of TGI (0.26 g Kg^−1^). This concentration was significantly higher than that obtained for RF-N by 126% and for MCERF-N by 319%. This increase in the concentration of the TGI compound in USRF-E indicates that the UVA-induced biosynthesis of phenolic compounds, and combined with UAE, enhances the extraction of this compound compared to the control (RF-N) and the MCE extraction method (MCERF-N). The use of UAE proved to be advantageous in the recovery of compounds such as PT (0.1–0.12 g Kg^−1^), BHDP (0.02 g Kg^−1^), SG (0.11–0.15 g Kg^−1^), and KCH (0.03–0.05 Kg^−1^). These compounds were not detected in the extract obtained through MCERF-N, indicating that the application of the ultrasound facilitated their extraction. Procyanidins and ellagitannins are bioactive compounds known for their potential health benefits, including anti-inflammatory, antioxidant, and anticancer properties [45,46]. These compounds are typically found in the floral organs of the strawberry plant and are present in low concentrations in the fruit itself [47].

The presence of agrimoniin as the primary compound in both ultrasound-assisted extraction (UAE) and maceration (MCE) extracts of strawberry agricultural by-products (RF) highlights its significance as a taxonomic marker of the *Rosaceae* family. Agrimoniin has gained importance in the nutraceutical market due to its bioactive properties, particularly its antioxidant potential [14]. In the case of RF with UAE and UVA treatments, the concentration of agrimoniin reached a maximum of 1.18 g Kg^−1^ in USRF-E, followed by 0.48 g Kg^−1^ in USRF-N and 0.1 g Kg^−1^ in MCERF-N. These concentrations were significantly higher than the reported concentration (0.14 g Kg^−1^) in strawberry fruit extracted with acetone [48]. Regarding ellagic acid derivatives, USRF-E showed a significantly higher concentration of EA (0.15 g Kg^−1^). At the same time, both USRF-N and USRF-E had similar (*p* < 0.05) concentrations of EAP (0.10–0.11 g Kg^−1^).

Among the flavonols, Q3G showed the highest concentration of 0.14 g Kg^−1^ in USRF-E, significantly higher than the reported concentration of 0.03 g Kg^−1^ in strawberries extracted with acetone [49]. The compound KCH (0.03–0.05 g Kg^−1^) was not detected in MCERF. The total sum of all quantified phenolic compounds (TPC_HPLC_) in RF, as shown in Figure 4, highlights the potential of RF as a valuable source of these bioactive compounds. By utilizing alternative technologies such as UVA radiation and ultrasound-assisted extraction, the concentration of phenolic compounds extracted from RF can be significantly enhanced, thereby increasing its added value. Specifically, the mean concentration of TPC_HPLC_ in USRF-E was 2.5 g Kg^−1^ RF, which is 85% higher than USRF-N and 400% higher than MCERF-N. Comparing these results with previous reports on strawberry agro-industrial by-products, the TPC_HPLC_ concentration obtained in USRF-E is similar to that reported for extraction with acetone (80%), at an extraction ratio of 1:10 *w/v*, and using an ultrasonic bath (1.42 g Kg^−1^) [4].

### 3.3. Digestive Stability of Phenolic Compound Extracted from Strawberry Agro-Industrial By-Products

Adequate and efficient extraction of phenolic compounds from secondary sources, such as RF, is crucial to scale up these processes and compete in the nutraceutical ingredients market. Nevertheless, a significant challenge associated with using phenolic-rich nutraceutical supplements is their low stability during gastrointestinal digestion. The extracts obtained under optimal UAE conditions were freeze-dried and underwent an in-vitro digestion process. The impact of the digestion process staged on the concentration of phenolic compounds was evaluated for both USRF-N and USRF-E, as presented in Table 4. It was observed that the various stages of the digestion process had a significant effect (*p* < 0.05) on the concentration of phenolic compounds.

During the salivary phase, most of the phenolic compounds in USRF-E exhibited significantly higher concentrations (*p* < 0.05) than in USRF-N. This result can be attributed to the UVA-induced biosynthesis of phenolic compounds in RF before extraction, which resulted in a higher initial concentration of phenolic compounds, apart from TGI and P3G. In the gastric phase, a significant reduction in the concentration of individual phenolic compounds was observed, with bioaccessibility ranging from 17.6% to 87.5% for USRF-N and 33% to 84% for USRF-E. The decrease in pH (1.3 ± 0.2) and the action of enzymes such as α-amylase and pepsin contributed to disrupting interactions and crystalline structures within the lyophilized extract, leading to the faster degradation of phenolic compounds. Despite the degradation, the trend of higher concentration in USRF-E continued, except for AE, which exhibited higher bioaccessibility in the gastric phase for USRF-N. Notably, the bioaccessibility of compounds SG and AGN in the gastric phase was higher in USRF-N (56.7% to 69.2%) with concentrations of 0.76 and 4.73 g Kg^−1^, respectively.

Nonetheless, these concentrations were lower than those of SG and AGN in the gastric phase of USRF-E (52.7% to 47.3%), with concentrations of 1.4 and 6.2 g Kg^−1^, respectively. Although USRF-E experienced a higher percentage loss during the gastric phase compared to USRF-N, the amount of SD and AGN that remained in the gastric fluid was greater for USRF-E.

In the intestinal phase, KCH and P3G were not identified or quantified in USRF-E, while in USRF-N, they remained stable at 28% and 25%, respectively. EA and Q3G exhibited the highest bioaccessibility in the intestinal phase, with values of 73.7% and 64% in USRF-E, similar to the bioaccessibility percentage reported by Kaeswurm et al. [50] for flavonols from seven apple varieties: 66% for flesh and 52% for peel. In terms of TPC_HPLC_, the number of phenolic compounds that remained stable in the gastric fluid and intestinal fluid was significantly higher in USRF-E (16.6 g Kg^−1^ to 10.5 g Kg^−1^) compared to USRF-N (9.8 g Kg^−1^ to 4.9 g Kg^−1^). Using UAE as an extraction technology provides technological advantages, such as reducing extraction times, increasing yields, and enhancing the bioaccessibility of phenolic compounds in RF. In a previously published study, the bioaccessibility of freeze-dried RF-N and RF-E was investigated without any extraction process, resulting in the bioaccessibility of 15.3% for RF-N and 22% for RF-E in the intestinal phase, with AGN being the major compound at a concentration of 0.45 g Kg^−1^ [13]. These values were lower than the concentrations reported for USRF-E in the intestinal phase (4.1 g Kg^−1^), highlighting the effect of the ultrasound in releasing phenolic compounds from the plant matrix, which otherwise hinders their solubilization and reduces their bioactive potential.

These findings highlight the significance of RF as a valuable source of ellagitannins. Ellagitannins have demonstrated their ability to interact with the microbiota of individuals and undergo biotransformation by bacteria such as *Gordonibacter urolithinfaciens*. This microbial process involves decarboxylation of an ellagic acid lactone ring, producing type A and B metabolites [51]. In-vivo studies have shown that these metabolites can enter the bloodstream within 6–8 h after consuming foods rich in ellagitannins [52]. These observations emphasize the potential health benefits associated with consuming ellagitannin-rich foods, such as RF, and their impact on gut microbial metabolism and systemic circulation of bioactive compounds.

### 3.4. Cell Culture Bioactivities of Ultrasound-Assisted RF (USRF) Extracts

#### 3.4.1. Anti-Proliferative Activity of Digested USRF Fractions

The sepals, stems, and peduncles that constitute RF are typically not consumed as part of the human diet. As a result, limited information is available regarding their nutraceutical potential and potential physiological interactions when utilizing RF as a potential nutraceutical supplement or ingredient. Consequently, it is essential to investigate the safety and bioactivity of the extracts derived from RF. Examining safety aspects, including toxicity evaluations and assessments of potential interactions with physiological processes, will contribute to a better understanding of the bioactivity and potential applications of RF extracts as nutraceutical supplements or ingredients.

**Figure 5:**
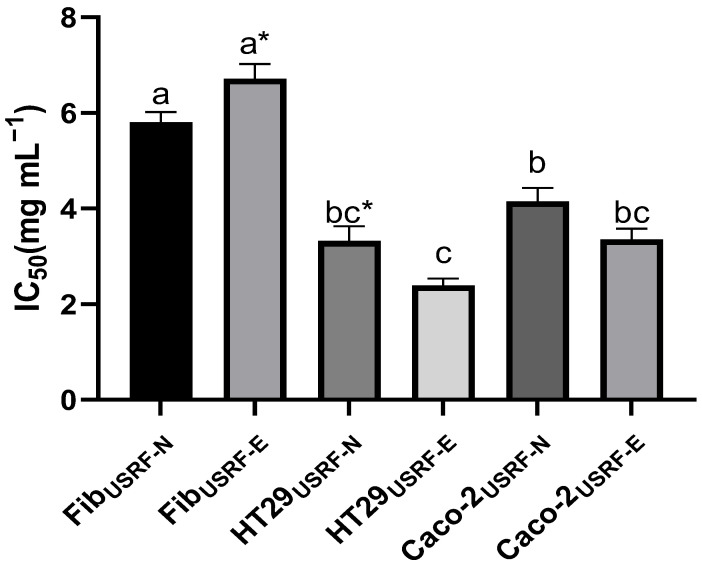
IC_50_ determination on healthy and cancer cells. Fib: Adult primary dermal fibroblast cell line. HT29: Human Colorectal Adenocarcinoma cell line. Caco-2: Human colorectal adenocarcinoma cell line. Different letter indicates significant differences between IC_50_ of different cell lines *p* < 0.05 by Tukey’s test. * Means, significant differences (*p* < 0.05) between the digested fraction of the optimal UVA-induced biosynthesis of phenolic compound and UAE extract (USRF-E) and its control (USRF-N) according to *t*-test (*p* < 0.05).

Figure 5 illustrates the IC_50_ values determined for the digested fractions of USRF-N and USRF-E in HT29 and Caco-2 colorectal cancer cell lines and a control group of healthy cells (HDFa, Fib). The IC_50_ for Fib was significantly higher for both FibUSRF-N (5.8 mg mL^−1^) and FibUSRF-E (6.7 mg mL^−1^) than the cancer cell lines. Among the cancer cell lines, HT29USRF-E exhibited the lowest IC_50_ value of 2.9 mg mL^−1^, indicating a lower concentration required to inhibit cell proliferation. This was followed by Caco-2USRF-N (4.1 mg mL^−1^) and Caco-2USRF-E (3.4 mg mL^−1^), with no significant difference observed between the latter two (*p* > 0.05). These findings demonstrate the potential anticancer properties of RF phenolic compounds and their potential safety, as the IC_50_ values in both colorectal cancer cell lines were lower than in healthy cells (Fib). Extracts obtained from UVA-treated strawberry by-products demonstrated a higher anti-proliferative capacity in the HT29 cell line. This phenomenon can be attributed, at least partially, to the enhanced bioaccessibility of phenolic compounds.

#### 3.4.2. Cellular Antioxidant and Anti-Inflammatory Activity

**Figure 6:**
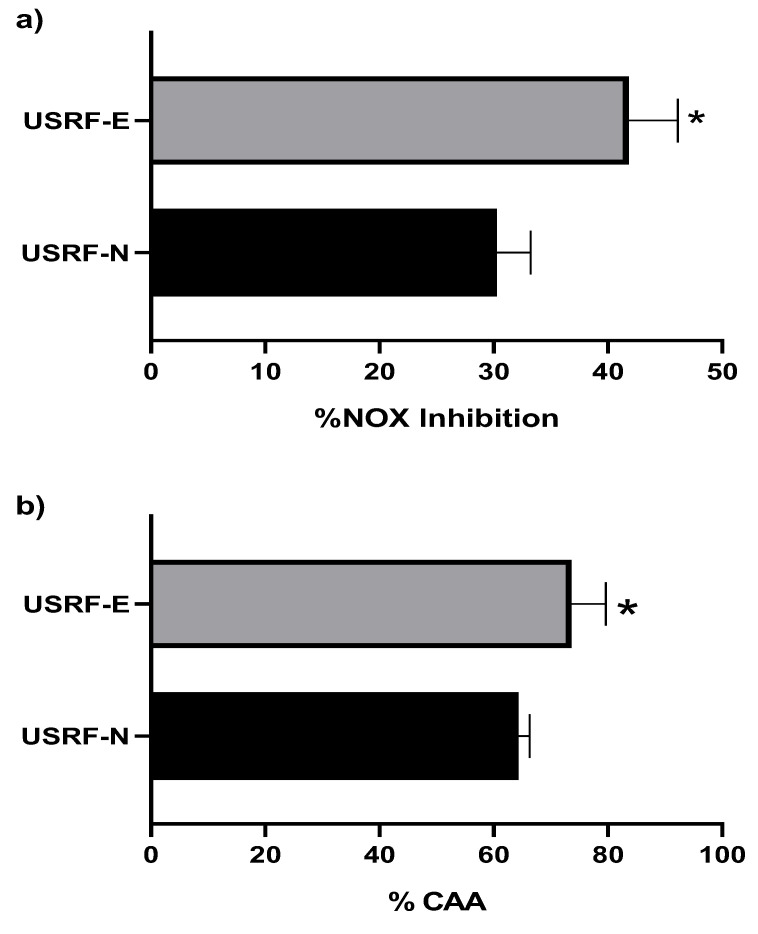
(**a**) Nitric Oxide (NOX) cell production inhibition (%). (**b**) Cellular antioxidant activity (%CAA). Bar indicates standard deviation. *: Means, significant differences (*p* < 0.05) between the digested fraction of the optimal UVA-induced biosynthesis of phenolic compound and UAE extract (USRF-E) and its control (USRF-N), according to *t*-test (*p* < 0.05).

Chronic degenerative diseases are characterized by a gradual decline in human health due to the alterations they induce in various tissues and organs of the body. One prominent hallmark of these diseases is the inflammatory response, which, when persistently activated, promotes disease progression, especially cancer [51]. A dose of 0.11 mg mL^−1^ was administered to evaluate the anti-inflammatory capacity of RF, which did not significantly affect the viability of RAW 254.7 cells (>80%). Figure 6a illustrates the results obtained regarding the inhibition of NOX production, comparing the digested fractions of USRF-N and USRF-E. It demonstrates that RF, treated with UVA radiation to enhance phenolic compounds followed by ultrasound-assisted extraction, significantly enhanced the anti-inflammatory potential of strawberry by-products by reducing NOX production by 42% with respect to the untreated group (control) and 12% higher than that exhibited by USRF-N (30%). This improvement can be attributed to the elevated presence of ellagitannins (1.0 g Kg^−1^ of EAP, 4.1 g Kg^−1^ of AGN, 1.2 g Kg^−1^ of TGI), as observed in Table 4, along with flavonols such as Q3G (1.1 g Kg^−1^) in the digested fractions of RF.

The UVA-induced biosynthesis of phenolic compounds not only promotes the accumulation of these compounds but also facilitates their interaction with cellular metabolism, leading to a reduction in proinflammatory signaling molecules like NOX. A study by Bibi et al. [53] demonstrated that a 0.1 mg mL^−1^ dose of raspberry extract, rich in ellagitannins and ellagic acid derivatives, increased the release of anti-inflammatory cytokines such as IL-10 and decreased levels of COX-2 and IL-1β. This result suggests that the RF extract may exert similar effects, highlighting its potential as a nutraceutical source, particularly for ellagitannins known for their potent anti-inflammatory activities in in-vivo studies [54].

Regarding the cellular antioxidant activity of the digested RF extracts, the inhibition of the APPH radical was measured. Figure 6b demonstrates that the digested fraction of USRF-E significantly delayed the pro-oxidant effect of the APPH radical in the Caco-2 cell line by 74%, higher than the digested fraction of USRF-N (64%) (*p* < 0.05). An imbalance between pro-oxidant and antioxidant species at the cellular level can indicate chronic degenerative diseases. Nonetheless, phenolic compounds can remarkably donate electrons, producing potent antioxidants in the plant kingdom. Regular consumption of these compounds in the human diet has been associated with controlling cellular oxidative stress [55]. The digested fraction of RF contains various classes of phenolic compounds, including ellagitannins, which can exhibit antioxidant effects by the oxidative ozone capacity of neighboring galloyl groups with free radicals [56].

Additionally, flavonols such as quercetin possess a high electron transfer capacity from hydroxyl groups in ring B, thereby regulating cellular oxidative stress [57]. This control of oxidative stress is crucial for reversing or preventing pre-disease states or metabolic alterations [55]. By utilizing these bioactive compounds, RF offers new sources of antioxidants currently being discarded, leading to adverse environmental impacts.

#### 3.4.3. Adipocyte Differentiation Inhibition Assay

**Figure 7:**
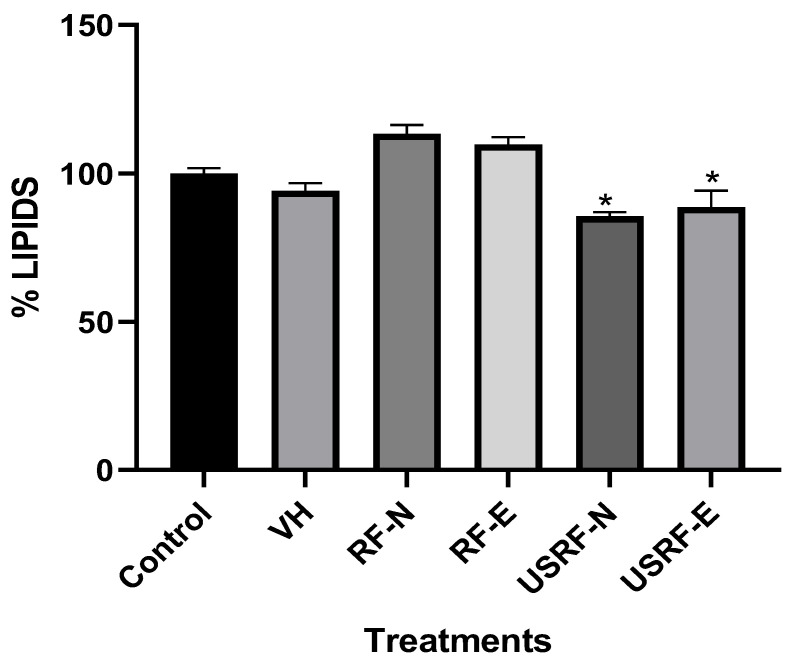
Inhibition effect of extracts of digested RF fractions on 3T3-L1 adipocyte differentiation. RF-N, Strawberry by-product control; RF-E, Strawberry by-product under optimal UVA-induced biosynthesis of phenolic compound; USRF-N, Ultrasound-assisted extract from strawberry by-product control; USRF-E, Ultrasound-assisted extract from strawberry by-product under optimal UVA-induced biosynthesis of phenolic compound *: Means, significant differences (*p <* 0.05) between the digested fraction and the control, according to *t*-test.

Adipocytes play a pivotal role in upholding lipid homeostasis and energy balance in vertebrates, either through triglyceride storage or the release of free fatty acids in response to fluctuations in energy demand [58]. Nevertheless, obesity is linked to several pathological conditions, including non-insulin-dependent diabetes, hypertension, hyperlipidemia, and cardiovascular disease [59]. Moreover, evidence suggests a direct correlation between triglyceride accumulation in skeletal muscles and pancreatic islets with insulin resistance in skeletal muscles and pancreatic β-cell dysfunction in obese individuals [60]. Obesity arises not only from adipose tissue hypertrophy but also from adipose tissue hyperplasia, which involves the conversion of preadipocytes into adipocytes. Therefore, it is imperative to explore alternative strategies that address the contemporary public health issue of obesity. Figure 7 displays the results obtained from differentiated 3T3 cells with lipid accumulation.

The anti-obesogenic activity of RF was evaluated using the digested fraction of strawberry agro-industrial by-products. The digested fraction was tested both with and without UVA-induced biosynthesis of the phenolic compound, without any additional extraction (RF-E and RF-E). This approach was consistent with a previous report [13]. The digested fractions were compared with the digested fractions of optimal extracts obtained using ultrasound assistance (USRF-N and USRF-E). Nonetheless, Figure 7 shows that the optimized extraction method significantly improved the capacity of the digested phenolic compounds to inhibit lipid accumulation in 3T3 cells, resulting in a notable reduction of 15% for USRF-N and 11.4% for USRF-E. These findings highlight the disparity in the potential consumption of RF without any extraction and emphasize the significance of effectively releasing these secondary metabolites from the plant matrix through solid–liquid extraction. This efficient extraction process positively contributes to the anti-obesogenic properties of phenolic compounds derived from such agro-industrial by-products.

Previous studies have reported that regular consumption of strawberries offers notable immunological benefits while contributing to weight control and addressing obesity concerns [61]. Nevertheless, the non-consumable parts of the fruit, such as RF, often end up in landfills, resulting in the loss of their bioactive potential for extracting phenolic compounds. Zhu et al. [8] conducted a study on the anti-adipogenic effect of the ethanolic extract of strawberries, including the stem and sepal, on 3T3 cells. They reported inhibition ranges between 23–39%, which were higher than the findings presented in this study.

Nevertheless, it is worth noting that the extract was directly applied in their study without undergoing any digestion process. As previously mentioned, the stability and phenolic compound content are significantly affected during digestion. Therefore, it is crucial to consider results that closely resemble the physiological conditions involving the phenolic compounds of RF. Ellagic acid and its derivatives play a fundamental role in this context, as they have been reported to possess an inhibitory potential on the expression of various genes and enzymes vital in lipid metabolism, including enzyme fatty acid synthase [62].

## 4. Conclusions

In conclusion, this study focused on the extraction, bioaccessibility, and bioactivity of phenolic compounds from strawberry agro-industrial by-products. Utilizing UVA-induced accumulation and ultrasound-assisted extraction (UAE), the RF extracts demonstrated high concentrations of phenolic compounds. The kinetic study of UAE highlighted the significant influence of the extraction ratio, ethanol percentage, and ultrasound power on the extraction yields of total phenolic content (TPC) from RF. Optimal conditions were identified with a 1:30 extraction ratio, 40% ethanol concentration, and 100% ultrasound power, resulting in an average TPC content of approximately 13 g GAE Kg^−1^, with agrimoniin as the major compound. The bioaccessibility study revealed the considerable impact of the digestion process on the concentration and bioavailability of phenolic compounds, with a maximum TPC_HPLC_ bioaccessibility of 36.5% for USRF-E.

Additionally, the RF extracts showcased promising bioactivity, including anticancer potential in colorectal cancer cell lines, anti-inflammatory properties through the reduction of NOX production, and cellular antioxidant activity by delaying the pro-oxidant effect of the APPH radical. Furthermore, the RF extracts demonstrated a reduction in adipogenesis. These findings underscore the nutraceutical potential of RF extracts and emphasize the significance of investigating their extraction, safety, bioactivity, and physiological interactions. The utilization of RF as a nutraceutical supplement or ingredient requires further exploration, considering its abundant ellagitannin content and potential interactions with the human microbiota. Moreover, these results highlight the ability of ultrasound technology for improving not only the extraction efficiency but also the bioactive properties of these compounds. By embracing such sustainable approaches, we can effectively tap into the potential of agro-industrial by-products such as RF, promoting environmental friendliness and economic viability within the agricultural and food industries.

## Figures and Tables

**Table 1:** Ultrasound intensity (UI) was calculated for each extraction condition tested.

Solvent	% US Power	UI (W m^−2^)
Water	20	264,243.70
Water	60	319,460.78
Water	100	316,666.67
EtOH 40%	20	195,665.73
EtOH 40%	60	208,713.49
EtOH 40%	100	195,462.65
EtOH 80%	20	129,973.05
EtOH 80%	60	135,099.69
EtOH 80%	100	140,890.20

US: Ultrasound. UI: Ultrasonic intensity. EtOH 40%: Ethanol–water mixture (40:60 *v/v*). EtOH 80%: Ethanol–water mixture (80:20 *v/v*).

**Table 2:** Box–Behnken design utilizing three variables alongside the determined and predicted values for Peleg’s rate constant (K1) and Peleg’s capacity constant (K2), derived from the total phenolic content kinetic model.

Run	Ratio (g mL^−1^) (X1: Coded value)	Ethanol Content (%*v/v*) (X2: Coded Value	Ultrasound Power (%) (X3: Coded Value)	K1 (min^−1^)		K2 (g GAE Kg^−1^)		
				Determined (Equation (1))	Predicted (Equation (8))	Determined (Equation (1))	Predicted (Equation (9))	Final Temperature °C
1	1:20(-1)	0(-)	60(0)	4.03	4.54	11.25	10.24	80
2	1:40(1)	0(-1)	60 (0)	4.87	4.76	9.86	10.75	77
3	1:20(-1)	80(1)	60 (0)	2.82	2.93	9.57	8.67	76
4	1:40(1)	80(1)	60 (0)	3.42	2.92	11.51	12.52	78
5	1:20(-1)	40(0)	20(-1)	3.07	2.22	11.31	13.38	67
6	1:40(1)	40(0)	20(-1)	6.96	6.71	12.63	12.79	62
7	1:20(-1)	40(0)	100(1)	7.21	7.46	12.41	12.25	71
8	1:40(1)	40(0)	100(1)	2.33	3.18	19.28	17.21	74
9	1:30(0)	0(-1)	20(-1)	5.44	5.78	12.48	11.50	76
10	1:30(0)	80(1)	20(-1)	1.14	1.88	10.46	9.29	82
11	1:30(0)	0(−1)	100(1)	5.20	4.45	9.64	10.82	75
12	1:30(0)	80(1)	100(1)	5.28	4.92	12.10	13.16	76
13	1:30(0)	40(0)	60(0)	5.79	5.27	14.16	14.35	77
14	1:30(0)	40(0)	60(0)	5.65	5.27	14.90	14.35	77
15	1:30(0)	40(0)	60(0)	4.37	5.27	13.98	14.35	77

**Table 3:** Peleg’s model coefficients for optimal total phenolic compounds extraction conditions.

Ratio (*w/v*)	%EtOH	%US	UI (W m^−2^)	Sample	Responses	K1	K2	adj-R^2^	RSME	TPC (g GAE Kg^−1^)
1:30	46.4	100	-	OPUSRF-N	Equations (8)–(9)	5.83	14.4	0.98	0.91	12.2 b
1:30	46.4	100	230,595.15	USRF-N	Equation (1)	7.71	12.53	0.98	0.47	11.4 ± 0.91 b
1:30	46.4	100	219,628.94	USRF-E	Equation (1)	7.37	13.77	0.98	0.56	12.9 ± 0.22 a
1:30	46.4	0	0	MCERF-N	Equation (1)	6.63	5.06	0.96	0.31	5.1 ± 0.19 d
1:30	46.4	0	0	MCERF-E	Equation (1)	4.99	6.04	0.94	0.42	6.1 ± 0.21 c

%EtOH: Ethanol percentage (*v/v*) in the solvent solution. %US: percentage power of ultrasound. OPUSRF: Ultrasound-assisted extraction of phenolic compounds from RF under optimal conditions. USRF-N: Ultrasound-assisted extraction of phenolic compounds under optimal conditions from strawberry by-products without UVA-induced phenolic compound biosynthesis. USRF-E: Ultrasound-assisted extraction under optimal conditions from strawberry by-products with UVA-induced phenolic compound biosynthesis. MCERF-N: Control phenolic compound extraction under optimal conditions without %US from RF-N. MCERF-E: Control phenolic compound extraction under optimal conditions without %USP from RF-E. K1: Peleg’s model rate constant. K2: Peleg’s model capacity constant. adj-R2: Adjusted regression coefficient. RSME: Root mean square error. Different lowercase letters indicate significant differences (*p* < 0.05) among the different extraction methods and RF condition, as determined by Tukey’s test.

**Table 4:** In-vitro digestion stability of phenolic compounds from strawberry by-products extract.

Sample	Compound		Concentration (g Kg^−1^)		Bioaccessibility (%)	
		Salival Fluid (SF)	Gastric Phase (GF)	Intestinal Phase (IF)	Gastric Phase (GF)	Intestinal Phase (IF)
USRF-N	TGI	2.24 ± 0.36 a	1.14 ± 0.32 b	0.55 ± 0.16 b	52.78	25.46
	PT	0.90 ± 0.10 a	0.37 ± 0.03 b	0.22 ± 0.10 c	38.95	23.16
	SG	1.14 ± 0.20 a	0.76 ± 0.13 b	0.22 ± 0.03 c	56.72	16.42
	PCT	0.88 ± 0.06 a	0.43 ± 0.02 b	0.29 ± 0.04 c	46.74	31.52
	BHDP	0.64 ± 0.09 a	0.06 ± 0.01 b	0.08 ± 0.003 b*	21.43	17.86
	EAP	0.76 ± 0.15 a	0.06 ± 0.004 b	0.06 ± 0.002 b	17.65	17.65
	AGN	5.40 ± 0.18 a	4.73 ± 0.43 a	2.52 ± 0.20 b	69.15	36.84
	EA	0.84 ± 0.03 a	0.57 ± 0.06 a*	0.16 ± 0.03 b	83.82 *	23.53
	Q3G	0.98 ± 0.03 a	0.51 ± 0.04 b	0.40 ± 0.03 b	36.43	28.57
	K3G	1.51 ± 0.48 a	0.88 ± 0.07 ab	0.30 ± 0.07 b	75.21	25.64
	KCH	0.41 ± 0.05 a	0.23 ± 0.04 b	0.10 ± 0.06 c*	65.71	28.57 *
	P3G	0.08 ± 0.03 a*	0.07 ± 0.04 a	0.02 ± 0.002 a*	87.50	25.00 *
	TPC_HPLC_	15.09 ± 0.96 a	9.82 ± 0.26 b	4.88 ± 0.25 c	59.84	29.74
USRF-E	TGI	2.15 ± 0.24 a	1.85 ± 0.22 a*	1.19 ± 0.15 b*	70.08 *	45.08 *
	PT	1.11 ± 0.04 a*	0.66 ± 0.02 b*	0.65 ± 0.02 b*	48.53 *	47.798 *
	SG	1.62 ± 0.16 a*	1.44 ± 0.20 a*	0.42 ± 0.01 b*	52.75 *	15.38 *
	PCT	2.41 ± 0.01 a*	1.62 ± 0.07 b*	0.66 ± 0.12 c*	72.00 *	29.33 *
	BHDP	0.63 ± 0.15 a	0.49 ± 0.02 a*	0.04 ± 0.001 b	58.33 *	4.76
	EAP	1.77 ± 0.08 a*	1.33 ± 0.10 b*	0.99 ± 0.02 c*	73.89 *	55.00 *
	AGN	8.13 ± 0.84 a*	6.21 ± 0.37 ab*	4.12 ± 1.20 c	47.30 *	31.38
	EA	1.42 ± 0.15 a*	0.25 ± 0.01 c	0.56 ± 0.02 b*	32.89	73.68 *
	Q3G	2.64 ± 0.02 a*	0.85 ± 0.16 c*	1.12 ± 0.08 b*	48.57 *	64.00 *
	K3G	1.98 ± 0.03 a	0.98 ± 0.05 b	0.70 ± 0.14 c*	71.53	51.09 *
	KCH	0.94 ± 0.004 a*	0.88 ± 0.07 b*	n.d.	83.81 *	n.d.
	P3G	0.04 ± 0.0001 a	0.02 ± 0.01 a	n.d.	33.33	n.d.
	TPC_HPLC_	24.82 ± 0.61 a*	16.6 ± 0.57 b*	10.45 ± 1.13 c *	57.94 *	36.47 *

USRF-N, Ultrasound-assisted extract from strawberry by-product control; USRF-E, Ultrasound-assisted extract from strawberry by-product under optimal UVA-induced phenolic compound biosynthesis. TGI, tetragalloyl glucose isomer; PT, procyanidin trimer; SG, saguinn; PCT, procyanidin tetramer; BHDP, bis-HHDP-glucose isomer; EAP, ellagic acid pentoxide; AGN, agrimoniin; EA, free ellagic acid. procyanidin tetramer; Q3, quercetin 3-o-glucuronide; K3G, Kaempferol 3-o-glucuronide; KCH, kaempferol-coumaroyl hexoxide; P3G, pelargonidin-3-o-glucuronide; TPCHPLC, Total phenolic content by HPLC. n.d., Not detected. Different lowercase letters in the same row indicate significant differences by Tukey’s test (*p* < 0.05). *: Means differences (*p* < 0.05) between the same compound of USRF-N and USRF-E samples.

## Data Availability

The data used to support the findings of this study can be made available by the corresponding author upon request.

## Supplementary Materials

The following supporting information can be downloaded at: https://www.mdpi.com/article/10.3390/foods12162989/s1, Table S1: Compositions of simulated gastrointestinal phases. Table S2. ANOVA for the influence of process condition on the kinetic parameters of Peleg’s model constant.

## References

[1] Ariza M.T., Reboredo-Rodríguez P., Cervantes L., Soria C., Martínez-Ferri E., González-Barreiro C., Cancho-Grande B., Battino M., Simal-Gándara J. (2018). Bioaccessibility and Potential Bioavailability of Phenolic Compounds from Achenes as a New Target for Strawberry Breeding Programs. Food Chem..

[2] Yang C., Lu J.-H., Xu M.-T., Shi X.-C., Song Z.-W., Chen T.-M., Herrera-Balandrano D.D., Zhang Y.-J., Laborda P., Shahriar M. (2022). Evaluation of Chitosan Coatings Enriched with Turmeric and Green Tea Extracts on Postharvest Preservation of Strawberries. LWT Food Sci. Technol..

[3] Priefer C., Jörissen J., Bräutigam K.R. (2016). Food Waste Prevention in Europe—A Cause-Driven Approach to Identify the Most Relevant Leverage Points for Action. Resour. Conserv. Recycl..

[4] Villamil-Galindo E., Van de Velde F., Piagentini A.M. (2020). Extracts from Strawberry By-Products Rich in Phenolic Compounds Reduce the Activity of Apple Polyphenol Oxidase. Lwt.

[5] Cano-Lamadrid M., Artés-Hernández F. (2021). By-Products Revalorization with Non-Thermal Treatments to Enhance Phytochemical Compounds of Fruit and Vegetables Derived Products: A Review. Foods.

[6] Campos D.A., Gómez-García R., Vilas-Boas A.A., Madureira A.R., Pintado M.M. (2020). Management of Fruit Industrial By-products—A Case Study on Circular Economy Approach. Molecules.

[7] Nowicka A., Kucharska A.Z., Sokół-Łętowska A., Fecka I. (2019). Comparison of Polyphenol Content and Antioxidant Capacity of Strawberry Fruit from 90 Cultivars of Fragaria × ananassa Duch. Food Chem..

[8] Zhu Q., Nakagawa T., Kishikawa A., Ohnuki K., Shimizu K. (2015). In Vitro Bioactivities and Phytochemical Profile of Various Parts of the Strawberry (*Fragaria × Ananassa* Var. *Amaou*). J. Funct. Foods.

[9] More P.R., Arya S.S. (2021). Intensification of Bio-Actives Extraction from Pomegranate Peel Using Pulsed Ultrasound: Effect of Factors, Correlation, Optimization and Antioxidant Bioactivities. Ultrason. Sonochem..

[10] Bengardino M.B., Fernandez M.V., Nutter J., Jagus R.J., Agüero M.V. (2019). Recovery of Bioactive Compounds from Beet Leaves through Simultaneous Extraction: Modelling and Process Optimization. Food Bioprod. Process..

[11] Girotto F., Alibardi L., Cossu R. (2015). Food Waste Generation and Industrial Uses: A Review. Waste Manag..

[12] Sánchez-Rangel J.C., Benavides J., Jacobo-Velázquez D.A. (2021). Valorization of Carrot Pomace: UVC Induced Accumulation of Antioxidant Phenolic Compounds. Appl. Sci..

[13] Villamil-Galindo E., Antunes-Ricardo M., Piagentini A.M., Jacobo-Velázquez D.A. (2022). Adding Value to Strawberry Agro-Industrial by-Products through Ultraviolet A-Induced Biofortification of Antioxidant and Anti-Inflammatory Phenolic Compounds. Front. Nutr..

[14] Villamil-Galindo E., Van de Velde F., Piagentini A.M. (2021). Strawberry Agro-Industrial by-Products as a Source of Bioactive Compounds: Effect of Cultivar on the Phenolic Profile and the Antioxidant Capacity. Bioresour. Bioprocess..

[15] Bustos-hipólito E., Legorreta-siañez A.V., Luisa A., Garfias J., González-gonzález L.R., Arenas-huertero F.J. (2012). Efecto de La Extracción de Los Compuestos Antioxidantes de La Cáscara de Manzana Con Solventes, Sobre La Bioactividad y Su Capacidad Antioxidante. Rev. Fac. Clencia y Tecnol. e Tecnol..

[16] Zielinski A.A.F., Haminiuk C.W.I., Beta T. (2016). Multi-Response Optimization of Phenolic Antioxidants from White Tea (*Camellia sinensis* L. Kuntze) and Their Identification by LC-DAD-Q-TOF-MS/MS. LWT Food Sci. Technol..

[17] Ones O., Rodriguez J. (2010). Evaluation of Physical Properties of Ethanolwater Mixtures. Rev. Fac. Ing. Univ. Antioq..

[18] Welti-Chanes J., Morales-de la Peña M., Jacobo-Velázquez D.A., Martín-Belloso O. (2017). Opportunities and Challenges of Ultrasound for Food Processing: An Industry Point of View. Ultrasound: Advances in Food Processing and Preservation.

[19] Milić P.S., Rajković K.M., Stamenković O.S., Veljković V.B. (2013). Kinetic Modeling and Optimization of Maceration and Ultrasound-Extraction of Resinoid from the Aerial Parts of White Lady’s Bedstraw (*Galium mollugo* L.). Ultrason. Sonochem..

[20] Urango A.C.M., Strieder M.M., Silva E.K., Meireles M.A.A. (2021). Thermosonication Process Design for Recovering Bioactive Compounds from Fennel: A Comparative Study with Conventional Extraction Techniques. Appl. Sci..

[21] Shorstkii I., Stuehmeier-Niehe C., Sosnin M., Mounassar E.H.A., Comiotto-Alles M., Siemer C., Toepfl S. (2023). Pulsed Electric Field Treatment Application to Improve Product Yield and Efficiency of Bioactive Compounds through Extraction from Peels in Kiwifruit Processing. J. Food Process. Preserv..

[22] Turk M.F., Baron A., Vorobiev E. (2010). Effect of Pulsed Electric Fields Treatment and Mash Size on Extraction and Composition of Apple Juices. J. Agric. Food Chem..

[23] Plazzotta S., Ibarz R., Manzocco L., Martín-Belloso O. (2020). Optimizing the Antioxidant Biocompound Recovery from Peach Waste Extraction Assisted by Ultrasounds or Microwaves. Ultrason. Sonochem..

[24] Hadidi M., Ibarz A., Pagan J. (2020). Optimisation and Kinetic Study of the Ultrasonic-Assisted Extraction of Total Saponins from Alfalfa (*Medicago sativa*) and Its Bioaccessibility Using the Response Surface Methodology. Food Chem..

[25] Galván D’Alessandro L., Dimitrov K., Vauchel P., Nikov I. (2014). Kinetics of Ultrasound Assisted Extraction of Anthocyanins from *Aronia melanocarpa* (Black Chokeberry) Wastes. Chem. Eng. Res. Des..

[26] Espinoza-Pérez J.D., Vargas A., Robles-Olvera V.J., Rodrı’guez-Jimenes G.C., Garcı’a-Alvarado M.A. (2007). Mathematical Modeling of Caffeine Kinetic during Solid–Liquid Extraction of Coffee Beans. J. Food Eng..

[27] Jurinjak Tušek A., Benković M., Belščak Cvitanović A., Valinger D., Jurina T., Gajdoš Kljusurić J. (2016). Kinetics and Thermodynamics of the Solid-Liquid Extraction Process of Total Polyphenols, Antioxidants and Extraction Yield from Asteraceae Plants. Ind. Crops Prod..

[28] Setford P.C., Jeffery D.W., Grbin P.R., Muhlack R.A. (2019). Mathematical Modelling of Anthocyanin Mass Transfer to Predict Extraction in Simulated Red Wine Fermentation Scenarios. Food Res. Int..

[29] González-Sarrías A., Núñez-Sánchez M.Á., Tomás-Barberán F.A., Espín J.C. (2017). Neuroprotective Effects of Bioavailable Polyphenol-Derived Metabolites against Oxidative Stress-Induced Cytotoxicity in Human Neuroblastoma SH-SY5Y Cells. J. Agric. Food Chem..

[30] Smeriglio A., Cornara L., Denaro M., Barreca D., Burlando B., Xiao J., Trombetta D. (2019). Antioxidant and Cytoprotective Activities of an Ancient Mediterranean Citrus (Citrus Lumia Risso) Albedo Extract: Microscopic Observations and Polyphenol Characterization. Food Chem..

[31] Abdillahi H.S., Verschaeve L., Finnie J.F., Van Staden J. (2012). Mutagenicity, Antimutagenicity and Cytotoxicity Evaluation of South African Podocarpus Species. J. Ethnopharmacol..

[32] Gutiérrez-Grijalva E.P., Antunes-Ricardo M., Acosta-Estrada B.A., Gutiérrez-Uribe J.A., Basilio Heredia J. (2019). Cellular Antioxidant Activity and in Vitro Inhibition of α-Glucosidase, α-Amylase and Pancreatic Lipase of Oregano Polyphenols under Simulated Gastrointestinal Digestion. Food Res. Int..

[33] Singleton V.L., Rossi J.A. (1965). Colorimetry of Total Phenolics with Phosphomolybdic-Phosphotungstic Acid Reagents. Am. J. Enol. Vitic..

[34] Flores F.P., Singh R.K., Kerr W.L., Pegg R.B., Kong F. (2014). Total Phenolics Content and Antioxidant Capacities of Microencapsulated Blueberry Anthocyanins during in Vitro Digestion. Food Chem..

[35] López-Barrios L., Antunes-Ricardo M., Gutiérrez-Uribe J.A. (2016). Changes in Antioxidant and Antiinflammatory Activity of Black Bean (*Phaseolus vulgaris* L.) Protein Isolates Due to Germination and Enzymatic Digestion. Food Chem..

[36] Wan H., Liu D., Yu X., Sun H., Li Y. (2015). A Caco-2 Cell-Based Quantitative Antioxidant Activity Assay for Antioxidants. Food Chem..

[37] Pacheco-Ordaz R., Antunes-Ricardo M., Gutiérrez-Uribe J., González-Aguilar G. (2018). Intestinal Permeability and Cellular Antioxidant Activity of Phenolic Compounds from Mango (*Mangifera indica* Cv. *Ataulfo*). Peels. Int. J. Mol. Sci..

[38] Krygowski T.M., Wrona P.K., Zielkowska U., Reichardt C. (1985). Empirical Parameters of Lewis Acidity and Basicity for Aqueous Binary Solvent Mixtures. Tetrahedron.

[39] Bosch E., Rosés M. (1992). Relationship between ET Polarity and Composition in Binary Solvent Mixtures. J. Chem. Soc. Faraday Trans..

[40] Santos-Zea L., Gutierrez-Uribe J.A., Benedito J. (2021). Effect of Solvent Composition on Ultrasound-Generated Intensity and Its Influence on the Ultrasonically Assisted Extraction of Bioactives from Agave Bagasse (*Agave salmiana*). Food Eng. Rev..

[41] Perez E.E., Carelli A.A., Crapiste G.H. (2011). Temperature-Dependent Diffusion Coefficient of Oil from Different Sunflower Seeds during Extraction with Hexane. J. Food Eng..

[42] Mudnic I., Modun D., Brizic I., Vukovic J., Generalic I., Katalinic V., Bilusic T., Ljubenkov I., Boban M. (2009). Cardiovascular Effects in Vitro of Aqueous Extract of Wild Strawberry (*Fragaria vesca* L.) Leaves. Phytomedicine.

[43] Pan Z., Qu W., Ma H., Atungulu G.G., McHugh T.H. (2011). Continuous and Pulsed Ultrasound-Assisted Extractions of Antioxidants from Pomegranate Peel. Ultrason. Sonochem..

[44] Ghafoor K., Choi Y.H., Jeon J.Y., Jo I.H. (2009). Optimization of Ultrasound-Assisted Extraction of Phenolic Compounds, Antioxidants, and Anthocyanins from Grape (*Vitis vinifera*) Seeds. J. Agric. Food Chem..

[45] Gesek J., Jakimiuk K., Atanasov A.G., Tomczyk M. (2021). Sanguiins—Promising Molecules with Broad Biological Potential. Int. J. Mol. Sci..

[46] Rue E.A., Rush M.D., van Breemen R.B. (2018). Procyanidins: A Comprehensive Review Encompassing Structure Elucidation via Mass Spectrometry. Phytochem. Rev..

[47] Hanhineva K., Rogachev I., Kokko H., Mintz-Oron S., Venger I., Kärenlampi S., Aharoni A. (2008). Non-Targeted Analysis of Spatial Metabolite Composition in Strawberry (*Fragaria × Ananassa*) Flowers. Phytochemistry.

[48] Aaby K., Mazur S., Nes A., Skrede G. (2012). Phenolic Compounds in Strawberry (*Fragaria × Ananassa* Duch.) Fruits: Composition in 27 Cultivars and Changes during Ripening. Food Chem..

[49] da Silva Pinto M., Lajolo F.M., Genovese M.I. (2008). Bioactive Compounds and Quantification of Total Ellagic Acid in Strawberries (*Fragaria × Ananassa* Duch.). Food Chem..

[50] Kaeswurm J.A.H., Burandt M.R., Mayer P.S., Straub L.V., Buchweitz M. (2022). Bioaccessibility of Apple Polyphenols from Peel and Flesh during Oral Digestion. J. Agric. Food Chem..

[51] Lopez-Corona A.V., Valencia-Espinosa I., González-Sánchez F.A., Sánchez-López A.L., Garcia-Amezquita L.E., Garcia-Varela R. (2022). Antioxidant, Anti-Inflammatory and Cytotoxic Activity of Phenolic Compound Family Extracted from Raspberries (*Rubus idaeus*): A General Review. Antioxidants.

[52] Milala J., Kosmala M., Karlińska E., Juśkiewicz J., Zduńczyk Z., Fotschki B. (2017). Ellagitannins from Strawberries with Different Degrees of Polymerization Showed Different Metabolism through Gastrointestinal Tract of Rats. J. Agric. Food Chem..

[53] Bibi S., Du M., Zhu M.J. (2018). Dietary Red Raspberry Reduces Colorectal Inflammation and Carcinogenic Risk in Mice with Dextran Sulfate Sodium-Induced Colitis. J. Nutr..

[54] Hou D.-X., Yanagita T., Uto T., Masuzaki S., Fujii M. (2005). Anthocyanidins Inhibit Cyclooxygenase-2 Expression in LPS-Evoked Macrophages: Structure–Activity Relationship and Molecular Mechanisms Involved. Biochem. Pharmacol..

[55] Forni C., Facchiano F., Bartoli M., Pieretti S., Facchiano A., D’Arcangelo D., Norelli S., Valle G., Nisini R., Beninati S. (2019). Beneficial Role of Phytochemicals on Oxidative Stress and Age-Related Diseases. Biomed Res. Int..

[56] Vogt T. (2010). Phenylpropanoid Biosynthesis. Mol. Plant.

[57] Hidalgo G.-I., Almajano M. (2017). Red Fruits: Extraction of Antioxidants, Phenolic Content, and Radical Scavenging Determination: A Review. Antioxidants.

[58] Frühbeck G., Gómez-Ambrosi J., Muruzábal F.J., Burrell M.A. (2001). The Adipocyte: A Model for Integration of Endocrine and Metabolic Signaling in Energy Metabolism Regulation. Am. J. Physiol. Endocrinol. Metab..

[59] Lee J., Jung E., Lee J., Kim S., Huh S., Kim Y., Kim Y., Byun S.Y., Kim Y.S., Park D. (2009). Isorhamnetin Represses Adipogenesis in 3T3-L1 Cells. Obesity.

[60] Tysoe O. (2021). Adipocytes Enter the Cell Cycle in Obesity. Nat. Rev. Endocrinol..

[61] Basu A., Izuora K., Betts N.M., Kinney J.W., Salazar A.M., Ebersole J.L., Scofield R.H. (2021). Dietary Strawberries Improve Cardiometabolic Risks in Adults with Obesity and Elevated Serum LDL Cholesterol in a Randomized Controlled Crossover Trial. Nutrients.

[62] Wu D., Ma X., Tian W. (2013). Pomegranate Husk Extract, Punicalagin and Ellagic Acid Inhibit Fatty Acid Synthase and Adipogenesis of 3T3-L1 Adipocyte. J. Funct. Foods.

