# Supplementary material for: Kinetic Ultrasound-Assisted Extraction as a Sustainable Approach for the Recovery of Phenolics Accumulated through UVA Treatment in Strawberry By-Products
Source: Foods. 2023 Aug 8;12(16):2989. doi: 10.3390/foods12162989 (PMC10453509; doi:10.3390/foods12162989)
Supplement: Supplementary file 1 [file foods-12-02989-s001.zip › foods-2533231-supplementary.pdf]

Supplementary Material

Table S1. Compositions of simulated gastrointestinal phases.

Reactive	Salival fluid (SF)	Gastric phase (GF)	Intestinal Phase (IF)	Duodenal Phase (DF)
**Deionized water (mL)**	500	500	500	500
**NaCl (g)**	0.0585	2.752	7.012	5.259
**KCl (g)**	0.0745	0.824	0.564	0.376
**$NaHCO_3$ (g)**	1.05		3.388	5.785
**Urea (g)**	0.2	0.085	0.1	0.25
**NaH_2PO_4 (g)**		0.266		
**$CaCl.{}_2H_2O$ (g)**		0.399		
**NH_4Cl (g)**		0.306		
**HCl (mL)**		6.5	0.18	0.15
**KH_2PO_4 (g)**			0.08	
**$MgCl_2$ (g)**			0.05	
**Enzymes**				
**α-Amylase (g)**	1			
**Pepsin (g)**		2.5		
**Pancreatin (g)**			9	
**Lipase (g)**			1.5	
**Bile Salt (g)**				8.2

Table S2. ANOVA for the influence of process condition on the kinetic parameters of Peleg's model constant.

Variation Source	DG	K1	K2
X1	1	*	*
X2	1	*	*
X3	1	*	*
X1*X2	1	ns	*
X1*X3	1	***	**
X2*X3	1	*	*
$X1^2$	1	ns	*
$X2^2$	1	*	***
$X3^2$	1	ns	ns
X1*X2*X3	1	ns	ns
$X1^2$*X2	1	ns	***
$X1^2$*X3	1	ns	ns
X1*$X2^2$	1	ns	ns
X1*$X3^2$	1	ns	**
$X2^2$*X3	1	ns	ns
X2*$X3^2$	1	ns	ns
R^2	-	0.87	0.99
Lack of Fit	14	ns	ns

X1: Solid-Liquid ratio (w/v). X2: Ethanol concentration (%v/v). X3: Ultrasound probe percent power (%). DG: degree of freedom. *: $p \leq 0.05$; **: $p \leq 0.01$; ***: $p \leq 0.001$. ns: $p > 0.05$.
